# Molekularpathologie bei urologischen Tumoren: Empfehlungen der Konsenskonferenz der Internationalen Gesellschaft für Uropathologie (ISUP) 2019

**DOI:** 10.1007/s00292-020-00888-4

**Published:** 2021-01-04

**Authors:** Oliver Hommerding, Yves Allory, Pedram Argani, Tarek A. Bismar, Lukas Bubendorf, Sofía Canete-Portillo, Alcides Chaux, Ying-Bei Chen, Liang Cheng, Antonio L. Cubilla, Lars Egevad, Anthony J. Gill, David J. Grignon, Arndt Hartmann, Ondrej Hes, Muhammad T. Idrees, Chia-Sui Kao, Margaret A. Knowles, Leendert H. J. Looijenga, Tamara L. Lotan, Colin C. Pritchard, Mark A. Rubin, Scott A. Tomlins, Theodorus H. Van der Kwast, Elsa F. Velazquez, Joshua I. Warrick, Sean R. Williamson, Glen Kristiansen

**Affiliations:** 1 Institut für Pathologie, Universitätsklinikum Bonn, Venusberg-Campus 1, Gebäude 62, 53127 Bonn, Deutschland; 2 Department of Pathology, Institut Curie, Saint-Cloud, Frankreich; 3 Department of Pathology, Johns Hopkins University School of Medicine, Baltimore, USA; 4 Department of Pathology and Laboratory Medicine, University of Calgary Cumming School of Medicine and Alberta Public Labs, Calgary, Kanada; 5 Institut für Medizinische Genetik und Pathologie, Universitätsspital Basel, Basel, Schweiz; 6 Pathology and Research Institute, National University of Asuncion, Asunción, Paraguay; 7 Department of Scientific Research, Norte University, Asunción, Paraguay; 8 Department of Pathology, Memorial Sloan Kettering Cancer Center, New York, USA; 9 Department of Pathology and Laboratory Medicine, Indiana University School of Medicine, Indianapolis, USA; 10 Pathology and Research Institute and Medical Sciences of National University of Asuncion, Asunción, Paraguay; 11 Department of Oncology and Pathology, Karolinska Institutet, Stockholm, Schweden; 12 Department of Anatomical Pathology, NSW Health Pathology, Sidney, Australien; 13 Department of Pathology, Indiana University School of Medicine, Indianapolis, USA; 14 Institut für Pathologie, Universitätsklinikum Erlangen, Friedrich-Alexander-Universität Erlangen-Nürnberg, Erlangen, Deutschland; 15 Medical Faculty and Charles University Hospital Plzen, Charles University, Pilsen, Tschechien; 16 Pathology, Indiana University School of Medicine, Indianapolis, USA; 17 Department of Pathology, Stanford University School of Medicine, Stanford, USA; 18 Divison of Molecular Medicine, Leeds Institute of Molecular Research at St James’s, St James’s University Hospital, Leeds, Großbritannien; 19 Princess Máxima Center for Pediatric Oncology, Utrecht, Niederlande; 20 Departments of Pathology, Oncology and Urology, Johns Hopkins University, Baltimore, USA; 21 Department of Laboratory Medicine, University of Washington, Seattle, USA; 22 Department for Biomedical Research, University of Bern and Bern Center for Precision Medicine, Bern, Schweiz; 23 Department of Pathology, Urology and Rogel Cancer Center, University of Michigan Medical School, Ann Arbor, USA; 24 Laboratory Medicine Program, University Health Network; Princess Margaret Cancer Centre, University of Toronto, Toronto, Kanada; 25 Inform Diagnostics and Tufts University, Boston, USA; 26 Department of Pathology, Penn State College of Medicine, Hershey, USA; 27 Department of Pathology and Laboratory Medicine and Henry Ford Cancer Institute, Henry Ford Health System, Detroit, USA; 28 Cancer Diagnosis and Pathology Research Group, Kolling Institute of Medical Research, Royal North Shore Hospital, St. Leonards, Australien; 29 Sydney Medical School, University of Sydney, Sydney, Australien; 30 Department of Pathology, Wayne State University School of Medicine, Detroit, USA

**Keywords:** Prostatakarzinom, Nierenzellkarzinom, Harnblasenkarzinom, Molekulare Biomarker, Zielgerichtete Therapie, Prostate cancer, Kidney cancer, Bladder cancer, Molecular biomarker, Targeted therapy

## Abstract

Das zunehmende Verständnis molekularer Grundlagen von Tumoren sowie der Fortschritt in der Diversifizierung der onkologischen Therapien versprechen individualisierte Therapieoptionen, welche bislang jedoch nur ansatzweise in die Therapieplanung von urologischen Tumoren eingegangen sind. Daher hat die Internationale Gesellschaft für Urologische Pathologie (ISUP) im März 2019 eine Konsenskonferenz zur Erarbeitung evidenzbasierter Handlungsempfehlungen zur molekularpathologischen Diagnostik beim Urothelkarzinom, Nierenzellkarzinom, Prostatakarzinom, Peniskarzinom und testikulären Keimzelltumoren durchgeführt. Die auf dieser Konsenskonferenz erarbeiteten Empfehlungen sind kürzlich in 5 separaten Manuskripten veröffentlich worden und werden in der vorliegenden Arbeit zusammengefasst.

Im Rahmen der Konferenzvorbereitung wurde eine umfassende Umfrage zur derzeitigen Praxis molekularer Testungen bei urogenitalen Tumoren unter den Mitgliedern der ISUP durchgeführt. Auf der Konferenz wurden die Ergebnisse und die entsprechenden Hintergrundinformationen durch 5 Arbeitsgruppen präsentiert und Handlungsempfehlungen für die Diagnostik erarbeitet. Eine Übereinstimmung von 66 % der Konferenzteilnehmer wurde als Konsens definiert.

## Abstract (en)

Comprehensive understanding of molecular principles in cancer and the diversification of oncological therapy promise individual therapeutic concepts, which have not yet found their way into urogenital cancer therapy. In March 2019 the International Society of Urogenital Pathology (ISUP) therefore held a consensus conference on recommendations for molecular diagnostics of genitourinary tumors, which were published in five separate manuscripts and are summarized in this article.

In preparation for the conference, a comprehensive survey of current practices for molecular testing of urogenital tumors was carried out by members of the ISUP. At the conference, the results and the corresponding background information were presented by five working groups and recommendations for action for diagnostics were developed. An agreement between 66% of the conference participants was defined as consensus.

## Urothelkarzinom

### Molekulare Subtypisierung, *TERT*- und *FGFR3*-Alterationen

Basierend auf der Expression von „luminalen/urothelialen“ und „basalen/squamösen“ Markern, der inflammatorischen Aktivierung und der Aktivität des Zellzyklus wurden in einer Konsensusklassifikation kürzlich 6 molekulare Subtypen des Urothelkarzinoms („luminal-papillär“, „luminal nicht spezifiziert“, „luminal instabil“, „stromareich“, „basal-squamös“ und „neuroendokrin-ähnlich“) definiert [37]. Der Großteil der muskelinvasiven Urothelkarzinome kann so entweder dem „luminalen“ oder dem „basal-squamösen“ Subtyp zugeordnet werden. Nur ein kleiner Teil wird aufgrund der Expression von neuroendokrinen Genen dem „neuroendokrin-ähnlichen“ Subtypen zugeordnet. „Luminal-papilläre“ Subtypen zeigen das längste (60 % nach 5 Jahren) und „neuroendokrin-ähnliche“ Subtypen das kürzeste Gesamtüberleben (15 % nach 5 Jahren) [57]. Die Datenlage bezüglich einer prädiktiven Wertigkeit der molekularen Subtypen im Hinblick auf das Ansprechen auf eine neoadjuvante Chemotherapie ist jedoch kontrovers und wurde an anderer Stelle in dieser Zeitung ausführlich diskutiert [55, 64, 66]. Zudem bestehen hohe technische Anforderungen bei Fehlen von etablierten immunhistochemischen Surrogatmarkerpanels, sodass die molekulare Subtypisierung noch keinen Eingang in die gegenwärtige Routinediagnostik gefunden hat.

Mutationen im Promotor des *TERT*-Gens, welches für eine katalytische Untereinheit der Telomerase codiert, kommen in etwa 60–80 % aller Urothelkarzinome vor und stellen eine frühe genetische Aberration in der Karzinogenese dar [38]. In benignen Läsionen wie reaktiven Urothelläsionen oder dem invertierten Papillom sind *TERT*-Mutationen nur sehr selten nachweisbar, so dass eine Mutationsanalyse in schwierigen Fällen hilfreich sein kann [32, 79].

Vor kurzem wurde in den USA der FGFR-Inhibitor Erdafitinib bei Patienten mit fortgeschrittenem/metastasiertem Urothelkarzinom zugelassen [71]. Als Voraussetzung müssen spezifische *FGFR3- oder FGFR2*-Alterationen nachgewiesen sein und ein Progress unter oder nach platinhaltiger Chemotherapie vorliegen. Parallel wurde eine Companion-diagnostic-Testung zur Detektion der *FGFR3- oder FGFR2*-Alterationen zugelassen [71]. *FGFR3*-Mutationen oder Translokationen finden sich in etwa 15 % der muskelinvasiven Urothelkarzinome, deutlich häufiger sind sie jedoch in den nichtinvasiven papillären Karzinomen (75 %) [57]. *FGFR3*-Alterationen sind bei nichtmuskelinvasiven Urothelkarzinomen mit einer geringeren Progressionsrate zum muskelinvasiven Urothelkarzinom und einer besseren Prognose assoziiert [74]. Es bestand Konsens, dass derzeit eine Integration des *FGFR3*-Status in die Tumorgraduierung oder die klinische Entscheidungsfindung verfrüht wäre. Eine Zulassung von Erdafitinib in Deutschland besteht zum aktuellen Zeitpunkt nicht (Stand Oktober 2020).

### Molekulare Biomarker in der Urinzytologie und Liquid-Biopsy-Diagnostik

Während für die Detektion eines High-grade-Urothelkarzinoms und eines Carcinoma in situ in der Urinzytologie eine hohe Sensitivität besteht, schließt ein negativer Befund ein Low-grade-Urothelkarzinom nicht aus. Vorteile uringebundener Biomarker liegen in der Erhöhung der Sensitivität für die Detektion von High-grade- und Low-grade-Urothelkarzinomen [31]. Erstere zeigen typische chromosomale Aberrationen wie Aneuploidien für die Chromosomen 3, 7, 17 sowie einen Verlust von 9p21, welche mittels FISH-Diagnostik nachgewiesen werden können (UroVysion-Test, Abbott Laboratories, Abbott Park, IL, USA) [15]. Für den molekularen Nachweis von Low-grade-Urothelkarzinomen können häufige und typische Genmutationen wie *TERT*- oder *FGFR3-*Mutationen herangezogen werden [11]. In der Nachsorge bestünde bei höherer Sensitivität der molekularpathologischen Untersuchungen das Szenario eines rein molekularen Rezidivs im Urin ohne Nachweis einer positiven Zytologie („antizipatorisch positiv“). Derzeit wird aber eine routinehafte Bestimmung uringebundener molekularer Biomarker in der Nachsorge nicht empfohlen.

Es gibt auch Fortschritte auf dem Gebiet der sog. Liquid-Biopsy-Diagnostik beim Urothelkarzinom. Der Nachweis von zirkulierenden Tumorzellen (CTCs) im Blut von Patienten mit Urothelkarzinom korreliert mit einem aggressiveren Krankheitsverlauf [8]. Dasselbe gilt für den Nachweis zirkulierender Tumor-DNA (ctcDNA) [20]. Daneben könnte zukünftig eine *FGFR*-Mutationsanalyse als Voraussetzung für eine Therapie mit Erdafitinib an ctcDNA durchgeführt werden. Trotz dieser vielversprechenden Entwicklungen bleibt die Liquid-Biopsy-Diagnostik vorerst auf Studien begrenzt.

### Seltene Histologische Varianten des Urothelkarzinom

Die Diagnose seltenerer histologischer Subtypen (mikropapilläres, plasmazytoides oder neuroendokrines Karzinom) ist relevant, da diese histologischen Varianten aggressiver sind, was ggf. auch ohne Nachweis einer Muskelinvasivität eine Frühzystektomie nahelegt.

Bei der plasmazytoiden Variante kommt es häufig zu einem E‑Cadherin-Verlust, welcher meist durch Mutationen im *CDH1*-Gen bedingt ist und das diskohäsive Wachstum dieser aggressiven Variante erklärt [3]. Eine E‑Cadherin-Immunhistochemie ist zur Diagnose nicht erforderlich, kann in Einzelfällen aber zur Unterscheidung von artifiziellem diskohäsivem Wachstum hilfreich sein.

Bei der mikropapillären Variante findet sich gehäuft (ca. 30 %) eine *HER2*-Amplifikation mit HER2-Überexpression, welche ggf. neue Therapieoptionen eröffnet [33]. Dennoch wird aufgrund der noch unzureichenden Datenlage vorerst keine routinehafte Testung empfohlen.

Das kleinzellige Karzinom der Harnblase, welches grundsätzlich anders therapiert wird, sollte mittels immunhistochemischer neuroendokriner Marker bestätigt werden. Da auch konventionelle Urothelkarzinome einen neuroendokrinen Immunphänotyp zeigen können, das Ansprechen auf eine neoadjuvante Chemotherapie für diese Tumoren aber unklar ist, sollten nur bei konventionell-morphologischem Bild eines kleinzelligen Karzinoms die immunhistochemischen Zusatzuntersuchungen erfolgen [70].

### Metastasiertes Urothelkarzinom und Immuntherapien

In etwa 20 % der Fälle findet sich beim lokal fortgeschrittenen und metastasierten Urothelkarzinom ein Therapieansprechen auf Checkpointinhibitoren, welche als Erst- und Zweitlinientherapie Einsatz finden [9, 69]. In Deutschland liegt eine Zulassung für Pembrolizumab und Atezolizumab in der Erstlinientherapie nur bei cisplatinungeeigneten Patienten vor, deren Tumorgewebe einen IC-Score von ≥5 % (Atezolizumab) oder einen CPS-Score von ≥10 (Pembrolizumab) aufweist. In der Zweitlinientherapie besteht zudem eine Zulassung für Nivolumab, wobei eine verpflichtende PD-L1-Immunhistochemie in der Zweitlinientherapie für alle 3 Therapeutika entfällt. Die ISUP empfiehlt eine routinehafte Testung des PD-L1-Status beim metastasierten Urothelkarzinom, übertragen auf Zulassungssituation in Deutschland ist entsprechend eine interdisziplinäre Absprache zu empfehlen.

Weniger als 1 % der Urothelkarzinome der Harnblase, aber etwa 20 % der Urothelkarzinome der oberen Harnwege sind hochgradig mikrosatelliteninstabil (MSI-high) bzw. Mismatch-Reparatur(MMR)-defizient. Letztere sind charakteristische urogenitale Tumoren im Rahmen des Lynch-Syndroms [12, 61]. In den USA ist durch die Food & Drug Administration (FDA) eine tumoragnostische Zulassung des Checkpointinhibitors Pembrolizumab für solide MSI-high oder MMR-defiziente Tumoren erfolgt [46]. Daher empfiehlt die ISUP eine routinemäßige Immunhistochemie für MLH1, PMS2, MSH2 und MSH6 bei allen Urothelkarzinomen der oberen Harnwege. Da eine tumoragnostische Zulassung für Pembrolizumab durch die Europäische Arzneimittel-Agentur (EMA) nicht umgesetzt wurde, ist eine Testung hier ebenfalls nur nach interdisziplinärer Absprache zu empfehlen.

## Peniskarzinom

### Stellenwert der p16-Immunhistochemie und der HPV-Testung beim Peniskarzinom und Vorläuferläsionen

Die penile intraepitheliale Neoplasie (PeIN) und das invasive Peniskarzinom werden in der WHO-Klassifikation basierend auf ätiologischen und prognostischen Merkmalen in die HPV(humanes Papillomavirus)-assoziierten und in die nicht-HPV-assoziierten Neoplasien unterteilt. Konventionell-morphologisches Charakteristikum der HPV-Infektion ist eine basaloide, kondylomatöse oder undifferenzierte Morphologie, während nicht HPV-assoziierte Neoplasien morphologisch meist verhornende Low-grade-Tumoren sind. Nahezu beweisend für eine High-risk-HPV-Infektion ist eine positive p16-Immunhistochemie, welche daher einen sensitiven Surrogatmarker darstellt [22].

Die morphologische Unterscheidung einer pleomorphen differenzierten PeIN von einer HPV-assoziierten PeIN mit hochgradiger intraepithelialer Neoplasie kann schwierig sein. Hier kann eine p16-Immunhistochemie zur Diagnose einer HPV-assoziierten PeIN mit hochgradiger intraepithelialer Neoplasie führen. Bei der Unterscheidung einer sog. hyperplasieartigen differenzierten PeIN von einer echten plattenepithelialen Hyperplasie kann eine Ki-67-Immunhistochemie helfen, da der Nachweis von suprabasaler Ki-67-positiver Zellen für eine hyperplasieartige differenzierte PeIN spricht.

Neuere Studien weisen auf das erhöhte Risiko einer malignen Entartung einzelner Subtypen von penilen Kondylomen mit High-risk-HPV-Infektion hin [26]. Daher wird empfohlen, eine p16-Immunhistochemie und/oder HPV-Typisierung von Kondylomen mit mäßig- und hochgradiger Atypie durchzuführen.

## Hodentumoren

Testikuläre Keimzelltumoren („testicular germ cell tumors“ [TGCT]) werden nach der derzeitigen WHO-Klassifikation basierend auf einer Assoziation mit einer Keimzellneoplasie in situ („germ cell neoplasia in situ“ [GCNIS]) in 3 Unterkategorien unterteilt. Das Fehlen oder Vorhandensein einer GCNIS muss nach Empfehlung der ISUP im Befund vermerkt werden.

Präpubertäre, nicht GCNIS-assoziierte Teratome (Typ-I-TGCT) sind benigne und treten hauptsächlich präpubertär, seltener auch postpubertär auf. Sie sind zytogenetisch diploid und weisen keine spezifischen genetischen Aberrationen auf [49]. Der Nachweis einer Aneuploidie, typisch als Verlust von Chromosom 6q, stellt das molekulare Korrelat der Transition in einen malignen, meist indolenten Dottersacktumor dar, welcher zusätzlich zum konventionell-morphologischen Aspekt über die Positivität für AFP und Glypican‑3 identifiziert werden kann [52]. Die Abgrenzung zu einem postpubertären, GCNIS-assoziierten malignen Teratom (Typ-II-TCGT) ist therapeutisch relevant und gelingt aufgrund identischer immunhistochemischer Profile nur über den Nachweis einer Oct3/4-positiven GCNIS im postpubertären, GCNIS-assoziierten Teratom. In schwierigen Fällen kann weiterhin der Nachweis einer Aneuploidie mit dem in GCNIS-assoziierten Teratomen vorkommenden Zugewinn an 12p zur Abgrenzung von einem präpubertären, nicht GCNIS-assoziierten Teratom genutzt werden [73]. Bei adoleszenten oder adulten Patienten sollte der konventionell-morphologische Verdacht auf ein präpubertäres, nicht GCNIS-assoziiertes Teratom mittels FISH für das Chromosom 12 bestätigt werden. Der Nachweis eines Zugewinns an Chromosom 12p kann bei unklaren primären oder metastasierten Tumoren weiterhin für die Bestätigung einer Keimzelltumoridentität dienen. Ausführliche Empfehlungen zur immunhistochemischen Diagnostik bei TGCT sind verfügbar [72].

Die Diagnose des spermatozytischen Tumors (Typ-III-TGCT) ist im Regelfall konventionell-morphologisch gut möglich. Bei diagnostischen Schwierigkeiten gelingt die Abgrenzung zum Seminom über eine negative Oct3/4-Immunhistochemie im spermatozytischen Tumor. Der im spermatozytischen Tumor regelhaft nachweisbare Zugewinn an Chromosom 9, welcher in Typ-II-TGCT nicht gefunden wird, hat in der Routinediagnostik keinen Stellenwert. Als Surrogatmarker für die letztgenannte Aberration kann jedoch eine Überexpression von DMRT1, welches auf Chromosom 9 lokalisiert ist, immunhistochemisch festgestellt werden [39].

Als vielversprechender Kandidat für einen Liquid-Biopsy-basierten molekularen Biomarker in der Primär- oder Verlaufsdiagnostik konnte die miRNA miR-371a-3p identifiziert werden [50]. miR-371a-3p wird von den malignen Komponenten aller TGCT, inklusive dem nicht GCNIS-assoziierten Dottersacktumor, überexprimiert und lässt sich, vorerst als experimenteller Marker, in Serum, Plasma und Liquor von Patienten nachweisen.

## Nierenzellkarzinom

Mit etwa 65–70 % stellt das klarzellige Nierenzellkarzinom (NZK) den häufigsten Subtypen aller NZK dar. Bekannte molekulare Veränderungen sind Mutationen oder Promotormethylierungen im *VHL*-Gen. Als „second hit“ findet sich typischerweise eine partielle oder komplette Deletion von Chromosom 3p [14]. Obgleich die Diagnose eines klarzelligen NZK häufig gut konventionell-morphologisch möglich ist, kann als Surrogatmarker für Alterationen in der VHL-HIF-Achse eine Carboanhydrase-9(CAIX)-Immunhistochemie eingesetzt werden. Nur eine starke, durchgängige membranöse Färbereaktion (analog zu einem HER2-Score von 3+) sollte als positiv gewertet werden. Zudem sollte beachtet werden, dass sich eine Positivität für CAIX generell auch in hypoxischen Geweben findet und eine nur perinekrotische Positivität nicht beachtet werden sollte.

Mutationen der ebenfalls auf Chromosom 3p lokalisierten Gene *SETD2, BAP1* und *PBRM1* sind offenbar auch mit dem biologischen Verhalten assoziiert, werden aber in der Routinediagnostik gegenwärtig nicht untersucht [53].

Das papilläre NZK stellt mit etwa 15–19 % den zweithäufigsten Subtypen aller NZK dar. Immunhistochemisch zeigen die Tumoren in den meisten Fällen eine Positivität für Zytokeratin 7 und AMACR. Bei Vorliegen multipler oder familiär gehäufter papillärer NZK Typ 1 ist an ein hereditäres papilläres NZK-Syndrom mit Keimbahnmutation im *MET*-Gen zu denken [62].

Das klarzellig-(tubulo-)papilläre NZK zeigt vornehmlich eine tubuläre, zystische und/oder papilläre Architektur mit klarzelligen Tumorzellen und uniformen, apikal ausgerichteten Zellkernen. Molekulare Charakteristika des klarzelligen oder des papillären NZK finden sich nicht, stattdessen sind Veränderungen der mitochondrialen DNA beschrieben [78]. Die Abgrenzung zum klarzelligen NZK ist aufgrund des indolenten Verhaltens relevant und gelingt über ein charakteristisches Immunprofil (Zytokeratin 7, GATA3 und CAIX [basolateral] positiv; AMACR und CD10 negativ) [59].

Die bisweilen schwierige Differenzialdiagnose eosinophiler bzw. onkozytärer Nierentumoren umfasst das renale Onkozytom (RO), das chromophobe Karzinom (ChRCC), die onkozytäre Variante des papillären NZK (OPRCC), die eosinophile Variante des klarzelligen NZK (CCRCC), den hybrid-onkozytisch-chromophoben Tumor (HOCT) sowie weitere, bisher unklassifizierte eosinophile Tumoren. Beim chromophoben Karzinom sind zytogenetische Veränderungen variabel und umfassen den Verlust an Chromosom Y, 1, 2, 6, 10, 13, 17 und 21 oder auch Gewinn an den Chromosomen 4, 7, 15, 19, und 20 [67]. Die 3 häufigsten molekularen Muster für das renale Onkozytom sind ein wildtypischer Karyotyp, ein Verlust von Chromosom 1 oder Y sowie Rearrangements von 11q13, welche das Gen für Cyclin D1 beinhaltet [4]. Weiterhin sind für das RO auch Verluste an Chromosom 1, X, Y, 14 oder 21 beschrieben. Die hybrid-onkozytisch-chromophoben Tumoren (HOCT) zeigen hierzu unterschiedliche genetische Profile [28].

Die in der WHO-Klassifikation als MiT-Familie der Translokationsnierenzellkarzinome zusammengefassten Entitäten beinhalten NZK mit Translokationen der Mitglieder der Mikrophtalmia-Transkriptionsfaktor-Familie *TFE3, TFEB* und *MiTF* [7]. Sie machen etwa 40 % der Nierenzellkarzinome bei pädiatrischen Patienten und bis zu 4 % der Nierenzellkarzinome im Erwachsenenalter aus. Neben den selteneren Translokationen von *TFEB* und *MiTF*, lokalisiert auf Chromsom 6 beziehungsweise Chromsom 3, stellen Translokationen von *TFE3*, lokalisiert auf Chromsom Xp11.2, die größte Untergruppe dar. Als Surrogatmarker für die vorliegenden Translokationen kann eine TFE3- oder TFEB-Immunhistochemie genutzt werden. Weiterhin werden regelhaft melanozytäre Marker (vornehmlich beim TFEB-assoziierten NZK) und Kathepsin K exprimiert [16, 47]. Hilfreich zur Abgrenzung eines klarzelligen NZK ist die negative oder schwache Färbereaktion für CAIX und die Keratinarmut dieser Tumoren [16]. An ein MiT-Translokations-Nierenzellkarzinom sollte bei einem NZK mit außergewöhnlicher Morphologie sowie bei jungen Patienten gedacht werden. Eine zusammenfassende Übersicht bezüglich klinischer, morphologischer, immunhistochemischer und molekularer Eigenschaften findet sich in der Originalarbeit (Tab. 2; [77]). Nach immunhistochemischem Nachweis von TFE3, TFEB und MiTF sollte bestätigend eine FISH-Untersuchung oder NGS-basierte Methode durchgeführt werden.

### Medulläres NZK

Das nahezu ausschließlich bei Patienten mit einer Sichelzellanämie oder seltener anderen Hämoglobinopathien vorkommende medulläre NKZ ist ein aggressives High-grade-Adenokarzinom mit infiltrativem, glandulärem Wachstumsmuster und desmoplastischer Stromareaktion. Ein Verlust der INI-1-Proteinexpression ist diagnostisch [35]. Medulläre Karzinome sollen, bei Fehlen einer Hämoglobinopathie, als *unklassifiziertes Nierenzellkarzinom mit medullärem Phänotyp* klassifiziert werden.

### Hereditäre NZK-Syndrome

Beim autosomal-dominant vererbten Von-Hippel-Lindau(VHL)-Syndrom liegen Keimbahnmutationen im *VHL*-Gen vor, welche für klarzellige NZK sowie extrarenale Neoplasien wie Hämangioblastome des zentralen Nervensystems, Phäochromozytome, Zystadenome des Pankreas und des Nebenhodens, neuroendokrine Tumoren sowie Nierenzysten prädisponieren. Der Verdacht auf das Vorliegen eines VHL-Syndroms sollte bei Nachweis eines klarzelligen NZK bei einem Patienten unter 46 Jahre oder bei Nachweis mehrerer klarzelliger NZK kommuniziert werden [65]. Konventionell-morphologisch zeigt sich als Besonderheit gegenüber den sporadischen klarzelligen NZK neben intratumoralen zystischen Tumoranteilen gelegentlich eine klarzellige papilläre Morphologie, welche aufgrund immunhistochemischer und zytogenetischer Merkmale jedoch als rein klarzelliges NZK klassifiziert werden sollte.

Succinat-Dehydrogenase(SDH)-defiziente Nierenzellkarzinome sind sehr selten [27]. Bei den meisten SDH-defizienten NZK liegen Loss-of-function-Keimbahnmutationen in der SDH-Untereinheit *SDHB* vor. SDH-defiziente NZK zeigen meist eine einheitliche Morphologie mit vakuoliertem, eosinophilem Zytoplasma mit Zytoplasmaeinschlüssen. Eine Positivität für Panzytokeratin fehlt in 25 % der Fälle. Als Surrogatmarker für die vorliegende SDH-Defizienz findet sich immunhistochemisch ein kompletter Expressionsverlust von SDHB. Hier gilt eine starke zytoplasmatische SDHB-Färbereaktion als erhaltene SDHB-Expression, angrenzende Tubuli sollten als interne Positivkontrolle verwendet werden. Nicht näher klassifizierbare eosinophile NZK, Zytokeratin-negative onkozytäre Tumoren und auch sarkomatoide Tumoren sollten weiter untersucht werden. Klinische Kriterien sind ein junges Patientenalter, multifokales Auftreten, eine positive Familienanamnese sowie das Vorliegen potenziell SDH-defizienter Neoplasien wie Paragangliome/Phäochromozytome, gastrointestinale Stromatumoren und Hypophysenadenome.

Beim autosomal-dominant vererbten Hereditäre-Leiomyomatose-und-Nierenzellkarzinom(HLRCC)-Syndrom liegen Keimbahnmutationen im Fumarathydratase-Gen vor [40]. Neben dem häufigen Auftreten von zahlreichen kutanen und uterinen Leiomyomen finden sich mit geringerer Penetranz NZK mit morphologischer Ähnlichkeit zu papillären NZK Typ II und insbesondere auffällig prominenten Nukleolen und perinukleolären Halos, welche ein wichtiges konventionell-morphologisches Kriterium darstellen. Als Surrogatmarker für die vorliegende FH-Defizienz findet sich immunhistochemisch ein kompletter Expressionsverlust der FH. Dieser ist jedoch nur in 80–90 % der FH-defizienten NZK nachweisbar, sodass bei begründetem Verdacht trotz positiver Färbereaktion für FH eine molekularpathologische Testung angeschlossen werden sollte.

### Aufkommende und provisorische Tumorentitäten

Bereits in der Vancouver-Klassifikation von 2012 waren neue, molekular definierte Nierenzelltumoren als aufkommende Tumorentitäten klassifiziert worden. Hierzu zählt das *ALK*-Translokations-NZK, bei welchem eine Fusion des anaplastischen Lymphomkinase(*ALK*)-Gens mit diversen Fusionspartnern zugrunde liegt [68]. Konventionell-morphologisch sind variable, meist cribriforme oder papilläre Wachstumsmuster beschrieben worden. Klinische Fallberichte zeigen ein Therapieansprechen auf den ALK-Inhibitor Alectinib [51]. Bei Verdacht auf ein *ALK*-Tanslokations-NZK empfiehlt sich die ALK-Immunhistochemie als Screeninguntersuchung, beweisend können eine *ALK*-FISH oder NGS-basierte Methoden eingesetzt werden.

## Prostatakarzinom

Mit dem stetig verbesserten Verständnis der molekularen Pathogenese des Prostatakarzinoms sind neue molekulare Biomarker mit prognostischer und therapieprädiktiver Wertigkeit entwickelt worden, welche bezüglich vorliegender Evidenz und einer möglichen Implementierung in die Risikostratifizierung auf der Konferenz diskutiert wurden.

### Prognostische Biomarker

Der Nutzen von prognostischen Biomarkern beim Prostatakarzinom liegt in der korrekten Differenzierung von letalen, heilbaren und insignifikanten Tumoren als Voraussetzung für die Erstellung einer individualisierten Therapie, idealerweise zum Zeitpunkt der initialen diagnostischen Biopsie. Insbesondere beim Therapiemanagement von Patienten mit niedrigem oder intermediärem Risiko besteht häufig Uneinigkeit, sodass neue molekulare Biomarker ebendort hilfreich wären.

Der Proliferationsmarker Ki-67 wird in Form des Ki-67-Proliferationsindex als diagnostischer und prognostischer Biomarker in verschiedenen Tumorentitäten bestimmt. Eine aktuelle Metaanalyse über 21 Studien mit insgesamt 5419 Patienten mit nichtmetastasiertem Prostatakarzinom belegte eindrucksvoll den Prognosewert des Ki-67-Proliferationsindex bezüglich des krebsspezifischen, des metastasenfreien Überlebens sowie des Gesamtüberlebens [10]. Mehrere Studien konnten zudem eine prognostische Wertigkeit des Ki-67-Proliferationsindex auch an Prostatanadelbiopsien bestätigen [36]. Nachteile des Ki-67-Proliferationsindex sind die hohe Interobservervariabilität und die Variabilität der Auswertungsmethode. Zudem sind zur Festlegung der Schwellenwerte für die Einstufung in eine Low-risk- oder High-risk-Kategorie weitere Studien notwendig.

Der Tumorsuppressor PTEN reguliert den onkogenen AKT-mTOR-Signalweg. Aberrationen des *PTEN*-Gens finden sich beim Prostatakarzinom in etwa 20 % der nichtmetastasierten sowie in 40 % der metastasierten Fälle [34]. Der *PTEN*-Status zeigt prognostische Wertigkeit für ein biochemisches Rezidiv und einen letalen Verlauf nach radikaler Prostatektomie [2, 41]. Der Nachweis eines *PTEN*-Verlustes im Biopsiematerial erhöhte das Risiko für eine Aufgraduierung am Prostatektomiepräparat [44], das frühere Auftreten eines kastrationsresistenten Prostatakarzinoms (CRPC), einer Metastasierung sowie einen tumorspezifischen Tod [48]. In sog. Active-surveillance-Kohorten war das Risiko eines Upgradings in der Rebiopsie bei *PTEN*-Verlust 2,6fach erhöht [42]. Eine prädiktive Wertigkeit für den *PTEN*-Status im Hinblick auf das radiografische progressionsfreie Überleben konnte in der aktuellen Phase-III-Studie IPATential150 gezeigt werden, in welcher der AKT-Inhibitor Ipatasertib in Kombination mit Abirateron und Prednisolon beim metastasierten CRPC (mCRPC) untersucht wird [24].

Zusammenfassend wurden der Ki-67-Proliferationsindex und der *PTEN*-Status als potenziell nützliche prognostische Biomarker bei der Evaluation einer aktiven Überwachung bei Patienten mit einem Prostatakarzinom mit ISUP-Graduierung 1 (und/oder ISUP-Graduierung 2) bewertet. Es bestand jedoch Konsens darüber, dass vor einer Empfehlung zur routinemäßigen Anwendung prospektive Studien zur Validierung und zum Vergleich mit alternativen Biomarkern notwendig sind.

#### RNA-basierte Biomarker

Genexpressionssignaturen stellen prognostische und prädiktive Biomarker beim lokal begrenzten Prostatakarzinom dar. Methodisch liegt ihnen eine Quantifizierung von mRNA mittels RT-PCR (Prolaris, Myriad Genetic Laboratories, Inc., Salt Lake City, UT, USA; OncotypeDx, Genomic Health, Inc., Redwood City, CA, USA) oder Mikroarray (Decipher, GenomeDX Biosciences, San Diego, CA, USA) aus FFPE-Gewebe zugrunde. Multiple Studien konnten für alle 3 Assays eine prognostische Wertigkeit belegen [13, 23, 60].

Abschließend bewertet die Arbeitsgruppe den gezielten Einsatz RNA-basierter Assays zur Abschätzung des Progressionsrisikos während einer aktiven Überwachung und nach radikaler Prostatektomie als prinzipiell sinnvoll. Bevor aber eine routinehafte Nutzung dieser kostenintensiven Assays empfohlen werden kann, sind jedoch weitere prospektive Validierungsstudien an Active-surveillance-Kohorten notwendig, in welchen diese auch mit etablierten und aktuell aufkommenden Biomarkern (wie z. B. Ki-67 oder PTEN) verglichen werden sollten.

### Prädiktive Biomarker: DNA-Reparatur-Defizienzen und Androgenrezeptoralterationen

Analog zu anderen Tumorentitäten konnten Defizienzen der homologen Rekombinationsreparatur (HRR) und der Mismatch-Reparatur (MMR) auch beim Prostatakarzinom als Prädiktoren für ein Therapieansprechen auf eine Chemo- oder Immuntherapie identifiziert werden.

In etwa 20 % aller fortgeschrittenen kastrationsresistenten Prostatakarzinome (CRPC) lassen sich Veränderungen in HRR-assoziierten Genen wie *BRCA1/2* und *ATM* nachweisen, von welchen etwa die Hälfte Keimbahnmutationen sind [58]. Eine signifikante Häufung von somatischen HRR-Mutationen bei metastasierten Prostatakarzinom (mCRPC) im Vergleich zum primären Prostatakarzinom lässt auf eine aggressivere Tumorbiologie der HRR-defizienten Malignome schließen [17]. Dies unterstützend sind HRR-Keimbahnmutationen mit aggressiven histologischen Varianten (duktales Adenokarzinom, intraduktales Karzinom des Prostata [IDC-P], Gleasonmuster 5) und letalen Krankheitsverläufen assoziiert [56, 63, 75]. In 2 retrospektive Studien werden Defizienzen der HRR als Prädiktor für das Ansprechen auf eine platinbasierte Chemotherapie beschrieben [19, 54]. Im Mai 2020 wurde in den USA und jetzt im November auch in Europa der PARP-Inhibitor Olaparib bei Patienten mit HRR-mutiertem mCRPC und Krankheitsprogress unter Abirateron und Enzalutamid zugelassen [25].

Mutationen in Genen der MMR-Proteine finden sich in bis zu 10 % aller CRPC und weniger als 3 % der primären Prostatakarzinome. Wie auch HRR-Mutationen sind MMR-Mutationen mit aggressiven histologischen Varianten (duktales Adenokarzinom, Gleasonmuster 5) assoziiert [30, 63]. Im Gegensatz zu HRR-Mutationen sind nur etwa 20 % der MMR-Mutationen Keimbahnmutationen. Studien weisen auf ein Therapieansprechen von MMR-defizienten CRPC mit Checkpointinhibitoren hin [6].

Zusammenfassend wird empfohlen, dass allen Patienten mit

- lokalisiertem Prostatakarzinom mit ISUP-Graduierung ≥4,
- lokalisiertem Prostatakarzinom aller ISUP-Graduierungen und einem PSA ≥20 ng/ml,
- oder metastasiertem Prostatakarzinom

eine HRR- und MMR-Mutationsanalyse der Keimbahn angeboten werden sollte, wenn dies klinisch angezeigt ist.

Eine HRR- und MMR-Mutationsanalyse an Tumorgewebe, präferenziell an Metastasengewebe, sollte allen Patienten mit metastasiertem Prostatakarzinom angeboten werden. Die Beurteilung einer MMR-Defizienz sollte eine Immunhistochemie für MLH1, PMS2, MSH2 und MSH6 mit oder ohne Analyse des MSI-Status und/oder Sequenzierung der MMR-Gene umfassen. Zur Beurteilung einer HRR-Defizienz sollte eine Sequenzierung zumindest von *BRCA1/2* mit Möglichkeit der Detektion vom Amplifikationen erfolgen.

### Androgenrezeptoralterationen

Genetische Aberrationen des Androgensrezeptors (AR) wie Punktmutationen, Amplifikationen des *AR*-Gens, *AR*-Splicevarianten (vor allem die *ARv7*-Splicevariante) und Amplifikationen von *AR*-Enhancer-Elementen führen zu konstitutiver Aktivierung des AR-Signalwegs unter Androgenablation und stellen das molekularpathologische Korrelat zur Kastrationsresistenz beim CRPC dar [29]. Neuere Wirkstoffe mit unterschiedlichen therapeutischen Ansatzpunkten wie der Reduktion der Androgenproduktion (Abirateron) oder der direkten Androgenrezeptorinhibition (Enzalutamid) stehen als therapeutische Optionen einer Taxan-basierten Chemotherapie gegenüber. Als potenzielle therapieprädiktive Biomarker sind die *ARv7*-Splicevariante in zirkulierenden Tumorzellen (CTCs) und *AR*-Amplifikationen in zellfreier DNA (cfDNA) untersucht. In einer retrospektiven Studie war der Nachweis der *ARv7*-Splicevariante in CTCs mit einer Therapieresistenz gegen AR-Signalweg-Inhibitoren assoziiert [5]. Zudem könnte der Nachweis von *AR*-Amplifikationen aus cfDNA als Prädiktor für das Ansprechen auf AR-Signalweg-Inhibitoren nützlich sein [21]. Bei Fehlen von prospektiven randomisierten Studien wird eine routinehafte Testung beim mCRPC gegenwärtig jedoch nicht empfohlen. Gewebebasierten Biomarkern kommen wegen nur schwacher prognostischer und fehlender prädiktiver Wertigkeit derzeit keine Bedeutung zu.

### Diagnostische Biomarker: neuroendokrines Prostatakarzinom (NEPC)

Die Abgrenzung primärer kleinzelliger neuroendokriner Prostatakarzinome (NEPC) und therapieassoziierter neuroendokriner Prostatakarzinome (t-NEPC) von Prostatakarzinomen mit fokaler neuroendokriner Differenzierung oder Karzinoiden ist wichtig und bisweilen schwierig. Hier gilt, dass eine kleinzellige Morphologie zur Diagnose eines NEPC erforderlich ist, da neuroendokrine Marker nicht spezifisch für das kleinzellige NEPC sind und fokal auch in konventionellen Adenokarzinomen gesehen werden. Genetische Aberration wie *RB*- oder *p53*-Inaktivierungen finden sich zwar gehäuft in NEPC, aber ebenfalls in konventionellen Adenokarzinomen, vor allem den CRPC [1]. Genomische Studien an CRPC zeigen eine Assoziation zwischen neuroendokriner Morphologie und neuroendokrinen Transkriptionssignaturen, diese ist allerdings nicht in allen Fällen gegeben [1]. Da eine fokale neuroendokrine Differenzierung mit höheren Gleason-Scores des gewöhnlichen Adenokarzinoms der Prostata assoziiert ist, ist bei diesen keine routinemäßige immunhistochemische Untersuchung neuroendokriner Marker empfohlen. Robuste therapieprädiktive Biomarker für das Ansprechen auf AR-Signalweg-Inhibitoren liegen für das fortgeschrittene CRPC nicht vor, zukünftig könnte hierzu eine Kombination molekularer und konventionell-morphologischer Merkmale herangezogen werden.
